# Supplementary material for: The Effect of Milk-Derived Extracellular Vesicles on Intestinal Epithelial Cell Proliferation
Source: Int J Mol Sci. 2024 Dec 17;25(24):13519. doi: 10.3390/ijms252413519 (PMC11678886; doi:10.3390/ijms252413519)
Supplement: Supplementary file 1 [file ijms-25-13519-s001.zip › Table S1.pdf]

Gene	Primer
Cyclin ***D1***	Forward: 5′-GCGGAGGAGAACAAACAGAT-3′<br>Reverse: 5′-TGAACTTCACATCTGTGGCA-3′
GAPDH	Forward: 5′-GCACCGTCAAGGCTGAGAAC-3′<br>reverse: 5′-ATGGTGGTGAAGACGCCAGT-3′

Table S1 Primers for mRNA detection.
