# Supplementary figures and images for: The Effect of Milk-Derived Extracellular Vesicles on Intestinal Epithelial Cell Proliferation
Source: Int J Mol Sci. 2024 Dec 17;25(24):13519. doi: 10.3390/ijms252413519 (PMC11678886; doi:10.3390/ijms252413519)

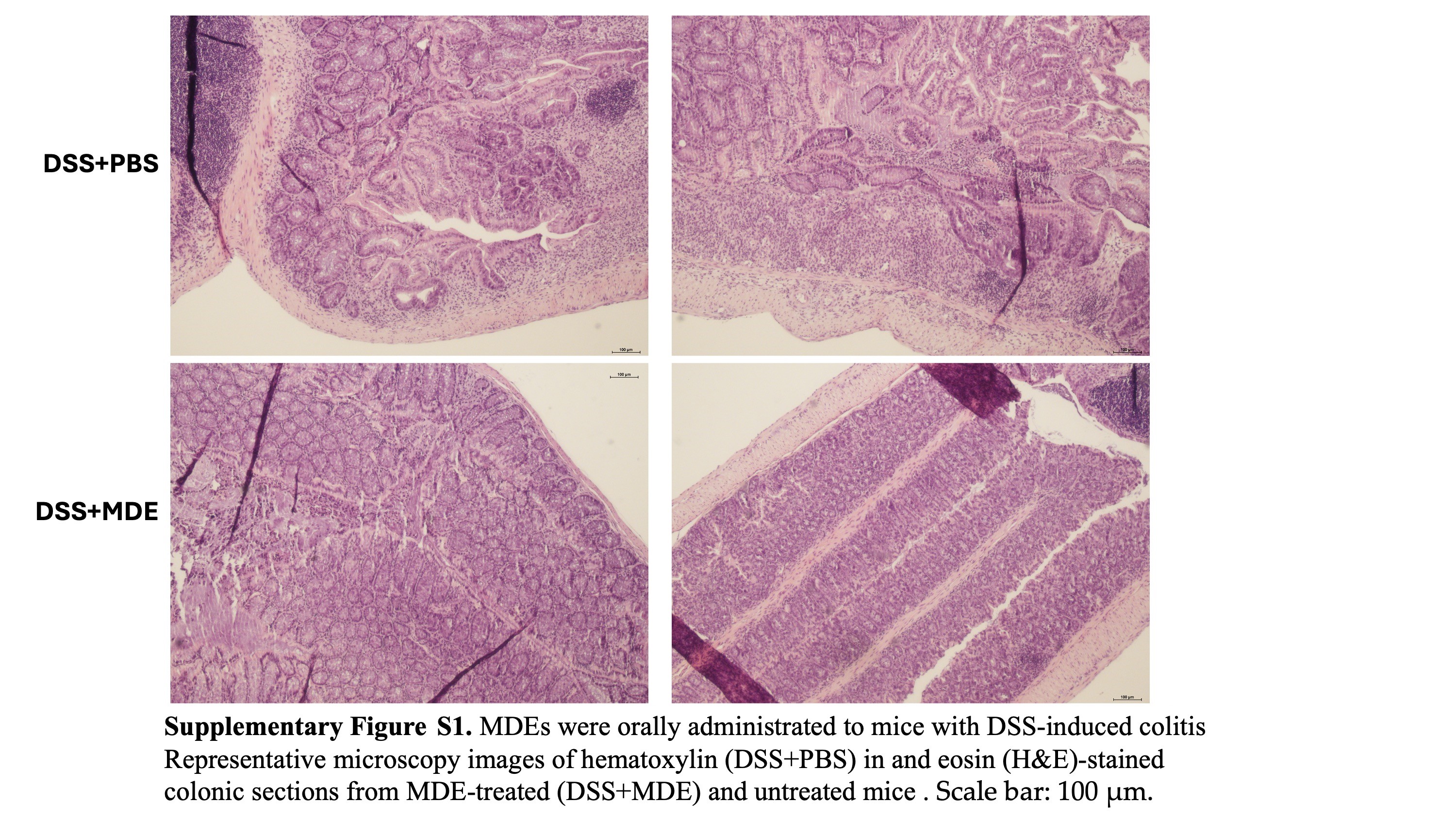

Supplement: Supplementary file 1 [file ijms-25-13519-s001.zip › Supplementary Figure S1.jpg]

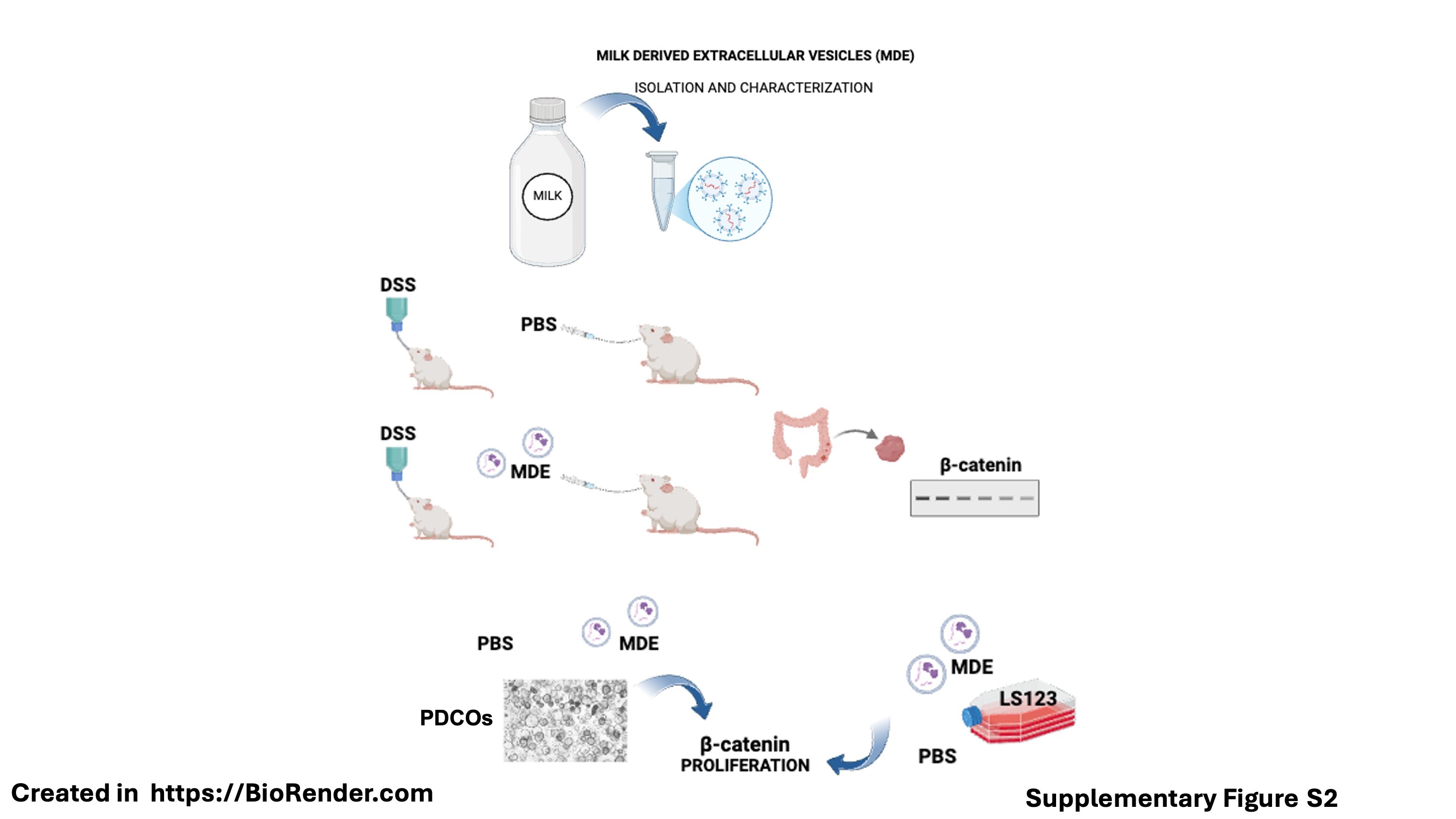

Supplement: Supplementary file 1 [file ijms-25-13519-s001.zip › Supplementary Figure S2.jpg]

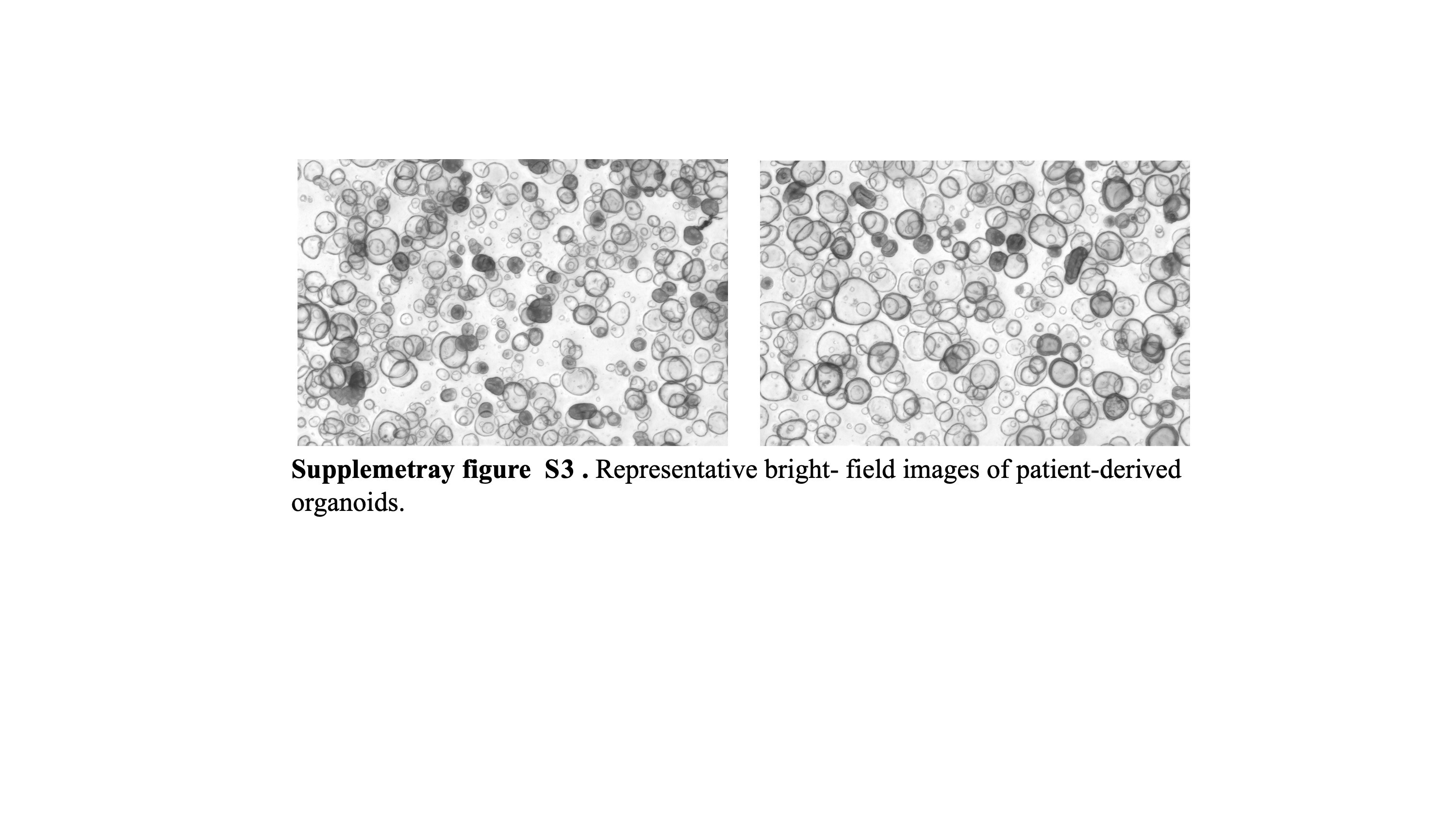

Supplement: Supplementary file 1 [file ijms-25-13519-s001.zip › Supplementary Figure S3.jpg]
